# A Qualitative Exploration of the Teaching- and Learning-Related Content Nursing Students Share to Social Media

**DOI:** 10.1177/08445621211053113

**Published:** 2021-11-10

**Authors:** Catherine M. Giroux, Katherine A. Moreau

**Affiliations:** 248180 ^1^Faculty of Education, University of Ottawa, Ottawa, Canada

**Keywords:** Nurse education, social media, qualitative approaches, technology, students

## Abstract

**Background:** Social media have many applications in health professions education. The current literature focuses on how faculty members use social media to supplement their teaching; less is known about how the students themselves use social media to support their educational activities. In this study, this digital artifact collection qualitatively explored what educational content nursing students shared with their social media accounts. **Methods:** A total of 24 nursing students’ Facebook, Twitter, and Instagram accounts were followed over 5 months. A modified directed content analysis was conducted weekly and at the end of the data collection period, using two cycles of inductive and deductive coding. **Results:** This study demonstrated that nursing students used social media to combat isolation, to consolidate course content, to share resources, and to better anticipate the transition to practice as a new nurse. **Conclusions:** Faculty members can capitalize on social media platforms to help nursing students explore nursing roles and identities while learning about and enacting professional online behaviours.

## Background and purpose

Health care professionals, students, and educators use social media in a variety of ways and for different purposes within health professions education (HPE). In a survey of medical educators, El Bialy and Jalali (2015) found that educators use social media to post opinions, share videos, chat, engage in medical education activities, take surveys, and play games. Other health profession educators select social media as a teaching tool because it aligns with program requirements. For instance, Tan et al. (2010) indicated that physiotherapy programs must provide evidence of reflective practice in their curriculum to meet professional standards; blogging provides a platform to achieve this educational requirement. Gagnon (2015) found that students use social media to communicate with classmates and instructors about academic work and share information related to courses and assignments.

Students also indicated that they use social media to network with health professionals and potential employers and to access articles and websites to use in clinical practice (Giordano & Giordano, 2011). Health care professionals reported using social media to share information, debate health care policy and practice issues, engage with the public, and for continuing education (Choo et al., 2015; Ventola, 2014). According to Choo et al. (2015), Twitter may also have psychological benefits for health care providers, allowing them to share discouraging experiences or professional challenges and gain feedback or validation from their peers. Finally, Batt (2016) found that 88% (n  =  219) of their health care professional survey respondents felt that self-directed activities like reading a blog, watching a webinar, or listening to a podcast constituted professional development activities.

Moreover, according to El Bialy and Jalali (2015) a major advantage of social media use in medical education is the creation of community. For example, hashtags like #ILookLikeASurgeon—which were created to address issues of sex stereotypes in surgery—have the power to unite individuals within and across specialty boundaries (Ovaere et al., 2018). Hills et al. (2016) extended this idea by introducing social media-based Communities of Practice (CoPs) in occupational therapy. The creation of virtual CoPs has become more prevalent with the advent of Twitter (Choo et al., 2015; Hills et al., 2016). Not only does the literature show that social media engage students and health professionals, but it also highlights the empowering nature of a connection to a worldwide online CoP (Choo et al., 2015; El Bialy & Jalali, 2015; Maloney et al., 2014; Tan et al., 2010). These CoPs help advance HPE by sharing links to learning resources and disseminating clinical pearls to trainees (Choo et al., 2015). For example, during the coronavirus-2019 (COVID-19) pandemic’s pivot to remote instruction, social media platforms offered spaces for faculty members to share and receive resources to facilitate the transition (Gottlieb et al., 2020; Prager et al., 2021).

Much of the focus of the nursing education literature at the undergraduate level is on the use of social media for teaching online professionalism to students (Barnable et al., 2018; Englund et al., 2012; Green et al., 2014; Marnocha & Pilliow, 2015). As with HPE generally, the literature on social media in nursing education commonly reported blogging for the purposes of reflection (Arbour et al., 2015; Chu et al., 2012; Garrity et al., 2014; Reed, 2012; Thomas et al., 2012). Social media was also used for virtual labs and simulations during the COVID-19 pandemic (Lebo & Brown, 2020). Another focus in the nursing literature is on using social media for exam preparation. Two studies indicated the benefits of using Facebook to practice exam questions collaboratively, like the National Council Licensure Examination (NCLEX) (Morales, 2017; Tower et al., 2014). Finally, Merriam and Hobba-Glose (2020) found that integrating social media platforms into online nursing courses increased students’ sense of emotional commitment and teaching presence.

In the Canadian nursing context, the Canadian Association of Schools of Nurses (CASN) identified core expectations for nursing programs, which relate to six domains: (a) knowledge; (b) research methodologies, critical inquiry, and evidence; (c) nursing practice; (d) communication and collaboration; (e) professionalism; and (f) leadership. Learners are encouraged and assisted to develop a broad knowledge base and to critically reflect on, integrate, and apply various forms of knowledge in diverse health care settings; Canadian baccalaureate nursing programs aim to prepare students for emerging information technologies and new approaches to patient safety, quality, and issues of global citizenship (CASN, 2016). According to CASN (2015), Canadian nursing students are expected to demonstrate several key competencies, including an appreciation for the importance of inquiry to the nursing profession; employ critical thinking skills to use relevant information and communication technologies to support evidence-informed nursing care; the ability to ensure client confidentiality and privacy, including in the context of social media; and the ability to advocate for change to address issues of social justice, health equity, and other disparities affecting population health. Social media could present an opportunity for nursing students to explore these competencies in their formal and informal learning.

Across the literature, many students and faculty members within HPE already use social media in various ways, including for personal and academic purposes (Gagnon et al., 2016; Giordano & Giordano, 2011; Laliberté et al., 2015). Much of the current literature quantitatively focused on which types of social media are used, by whom, and how often. Studies focused on, for example, the use of social media at conferences or its use for professional networking or engaging with the public. Very few studies explored what content nursing students share with social media for their formal learning (i.e., related to courses and assignments) and informal learning (i.e., related to extracurricular activities or personal learning goals), if they share anything at all. To address this gap, this study used a digital artifact collection to explore the question of what content students in a school of nursing post to social media related to formal and informal learning.

## Methods and procedures

### Research design and data collection

The present study represents a 5-month-long digital artifact collection that explored how nursing students at one Canadian school of nursing used social media in their learning. The term *digital artifacts*, in this context, referred to the content that nursing student participants posted to their social media accounts including but not limited to videos, pictures, memes, text, news articles, academic articles, and personal comments and reflections. The term *post* refers to the original content nursing students added to a social media platform. The term *share* refers to the students circulating pre-existing posts on a social media platform (e.g., retweeting, sharing a Facebook post). Nursing students could have also added their own unique posts to shared social media content. For instance, they may have chosen to post their perspective or comments on a news article they shared.

We requested permission to follow nursing students on social media using accounts designed specifically for this project. Participants provided their social media handles for us to follow and their acceptance of our request signified their consent to participate in this study. A total of 24 nursing students from the school of nursing permitted us to follow their social media accounts. We observed participants’ social media accounts for a 5-month period, from March to August 2019. As a mechanism to prevent any Hawthorne effect (i.e., participants changing their posting behaviour since they were aware they were being observed), we chose to observe participants over an extended period with the goal of capturing consistent posting behaviour over this 5-month period. We did not interact with any participant posts on social media to maintain our position as observers.

We recorded notes on a social media data extraction form at the end of every week during this 5-month period. These notes focused on the nature and content of the social media postings shared by participants, specifically the learning elements, images, text, sounds, music, gestures, and use of space in the posting (Machin, 2007). We did not collect any identifiable information (e.g., names, names of institutions, photos, videos) and did not include any posts or comments shared by participants’ friends or followers who had not consented to participate in this study.

### Data analysis

An ongoing modified directed content analysis of the data extraction form occurred at the end of every week of the digital artifact collection period (Hsieh & Shannon, 2005). An initial round of coding occurred at the end of each month, using a combination of deductive coding with a codebook and inductive coding to identify new codes. The literature review, research questions, and CASN’s National Nursing Education Framework (2015) informed our codebook. A process of monthly analytic memoing detailed subcategories that arose in the participants’ social media posts and noted our reflections on any surprising findings to enhance our researcher reflexivity (Yin, 2014). MAXQDA (v.18.2) facilitated a modified directed content analysis on the entire data set at the end of the 5-month data collection period, using two cycles of inductive and deductive coding. We engaged in peer-debriefing throughout the data analysis process to ensure trustworthiness of our analysis and interpretations (Guba, 1981; Lincoln & Guba, 1985).

### Ethical considerations

We met with a member of our institutional Research Ethics Board (REB) in March 2018 to clarify what was required ethically for the digital artifact collection. The REB determined that any public social media posts (e.g., a public Twitter account) did not require us to obtain consent from participants before collecting data. However, any social media posts we accessed from private or personal social media accounts required us to send participants a Participant Information Letter (PIL) and required them to provide informed consent to participate in the study. The REB also determined that beyond sending participants a PIL, we would send them a request for our research social media accounts to ‘friend’ or follow their personal social media accounts. Participants’ acceptance of our request to ‘friend’ or follow them constituted informed consent to participate in the digital artifact collection. Participants had the opportunity to terminate their participation in the study at any point by notifying us through email or by ‘unfriending’ or unfollowing our research accounts.

This digital artifact collection received formal institutional ethical approval (S-08-18-921) and approval from the study site (101916) in August 2018.

## Results

Of the 24 nursing students who participated in the digital artifact collection, 15 provided Facebook information, five provided Twitter handles, and 19 provided Instagram handles. Facebook received the largest proportion of posts related to learning in nursing by students while Twitter and Instagram received fewer. The number of posts per platform varied by month. Table 1 provides an overview of the number of posts that nursing students made related to learning in nursing by platform by month during the 5-month data collection period.

**Table 1.** Social Media Posts Related to Learning by Platform by Month (n  =  24).

Social Media Posts by Platform by Month				
	Facebook	Instagram	Twitter	Total
March	19	5	2	26
April	55	10	3	68
May	48	5	6	59
June	51	5	6	62
July	42	4	4	50
August	31	4	8	43
**Total**	246	33	29	**308**

The participants posted content related to learning in nursing to social media at differing frequencies. Table 2 depicts the frequencies with which participants posted content related to learning in nursing education online. Note that seven participants consented to participate in the digital artifact collection but did not post any public-facing content related to learning in nursing to their personal social media pages during the 5-month data collection period.

**Table 2.** Frequency of Posts Related to Learning in Nursing Education by Participant (n  =  24).

Number of Posts	Frequency
178	1
43	1
26	1
14	1
9	1
8	1
7	1
5	1
4	1
3	3
1	5
0	7

Since all participants consented to participate in the digital artifact collection and we followed their social media accounts for the duration of the 5-month data collection period, we did not exclude any participants despite the fact that seven of them did not share content related to learning in nursing. That participants chose not to share nursing-related content on their personal social media accounts is a finding in itself. These seven participants did share personal posts, which were not included in the study, to their social media accounts during the data collection period.

The nursing students who participated in this study posted diverse content to their Facebook, Twitter, and Instagram accounts, including 78 articles, 54 memes, 56 original posts, and 120 posts from peers or organizations that related to formal or informal learning in nursing and touched on several of the essential components for baccalaureate nursing education identified by CASN (2015). This content related to four overarching categories, which included advocacy; nursing identity, socialization, and culture; formal and informal learning in nursing; and sharing educational tools, jobs, and resources.

### Advocacy

Several nursing students posted contemporary political and public health-related advocacy content. The advocacy topics varied by month and often aligned with major topics and issues that were occurring in the news. As such, many of the posts contained links to news articles as stand-alone posts but often also included the students’ commentary on the issues. A major subcategory that spanned the entire 5-month data collection period pertained to advocacy surrounding vaccinations. Students shared news articles about measles outbreaks and bans on unvaccinated people entering certain countries or districts. Other students shared posts that illustrated the historical development of vaccines. Another source of advocacy content related to nursing culture and working conditions. Many of these posts occurred in April and May, when Republican Senator Maureen Walsh from Walla Walla made the statement that nurses should not be allowed to work 12-h shifts—instead of the 8 h shifts they were currently working—because then they would “just spend more time complaining of being tired and playing cards.” Several students posted original posts or shared memes related to this topic and reiterating the fact that nurses do not play cards. Other articles that students shared related to nurses who face violence in the workplace, rallies in Quebec to end forced overtime, and not having enough time or staff to properly care for patients, especially in long-term care settings.

The nursing students also posted about mental health and addictions frequently. Several students shared Facebook posts that indicated the signs and symptoms of a drug overdose and encouraged their friends/followers to carry Narcan kits. Other students shared news articles from local newspapers of youth who had died by overdose. One participant added:

I cannot stress this enough. If your kids even smoke pot, be sure they have Narcan kits nearby. Fentanyl is being found in marijuana, molly, fake oxy, knock off percs, cocaine, and pot edibles. While it may be safe as prescribed by doctors, concentrations cannot be controlled outside legit manufacturers and it kills people by interrupting the body’s natural signals to keep breathing. People do not go into distress, they appear calm, mellow, and high. Would you know the difference between a high and imminent death? Please talk to your kids. Again. And again. Nagging doesn’t kill people. (Participant 08)

A number of posts related to the opioid crisis, including several news articles that described the impacts of the opioid crisis on both health policy and health delivery.

### Nursing identity, socialization, and culture

This second category of postings related to the topic of nursing identity, socialization, and culture. As with the category of advocacy, the nursing identity posts varied by month, depending on what events appeared in the news media during that time period. Interestingly, a number of students identified themselves as nurses or nursing students in their social media biographies. A total of 11 students shared their nursing student/nurse status in their Instagram bios, five students included this information in their Facebook profiles, and two students shared their nursing student/nurse status in their Twitter bios.

A prominent subcategory related to how nurses were perceived on social media. Some students posted memes and videos that highlighted common perceptions or stereotypes of nursing personalities. Several participants shared posts from others that highlighted tender moments between nurses and their patients. One student shared a post from a page called ‘I Love Nursing’ that described how “nursing is the strange world where you check out entirely from your own life to dive head first, knee deep into the drama, struggles, challenges, achievements, successes, and deepest darkest truths of complete strangers 12 h at a time. Then back to your regular life.” Students also posted content that depicted what life was like for nurses both inside and outside of their professional responsibilities.

One student shared a post that had originally been posted to Facebook by someone else. The post describes aspects of nursing that are often unseen by patients and families, like how nurses often have to deal with trauma and resuscitation and then move along to the next patient without any down time. The post urges patients to be mindful of the things they do not see in their health care encounters. Similarly, a student retweeted a tweet that reads “One of the many take away points while furthering my education: Nursing is hard. It is, however, harder than it needs to be for reasons beyond a nurse’s control… which sucks.” The student added, “Just talked about this today at work. So much of what we encounter daily we have zero control over. Which equals burnout, higher sick calls & staff turnover, and poorer patient outcomes. Everyone needs to change this because everyone benefits” (Participant 08).

Finally, other posts related to challenging patient encounters or tension between nurses and patients. One student shared a meme that featured Leonardo DiCaprio holding up a martini glass at a party. The meme read “I know you’re lying, continue.” The student added the hashtags ‘my day’ and ‘nurse life’. Another meme related to tension between patients and nurses read “the patient who googles all of their signs and symptoms before getting to the hospital.” The meme featured Homer Simpson lying on his back on a couch smoking a cigar. It read “everyone is stupid except for me.” The participant added, “and THEN we ask: why are you here?” Another similar meme featured a man appearing to lecture a brick wall. The meme reads, “how educating a non-compliant patient looks like.”

### Formal and informal learning in nursing

Numerous students shared artifacts related to their specific nursing courses or their semesters more generally. The 5-month digital artifact collection covered the end of the Winter 2019 semester, the start and end of the Spring 2019 and Summer 2019 semesters (if applicable), and the start of the Fall 2019 semester. As a result, participants shared images of themselves celebrating the end of courses in the Spring. Others shared memes related to the amount of work they had to do in a short space of time. Students shared posts relating to their clinical placements. Participant 06 shared an Instagram story of them taking a mirror selfie. They are wearing black scrubs and smiling at the camera. The caption on the photo read, “happy to be in scrubs for the day.” It also included a cartoon heart image with ECG (electrocardiogram) tracings over top. The same student posted an image of nine people standing in grey t-shirts and black pants in front of a large map displaying the coordinates of the location of their clinical placements. They are all wearing hospital ID cards. The student posted the caption, “Thanks for a great semester team” along with a celebration emoji. Participant 14 similarly shared a picture of a group of seven people wearing black scrubs, white running shoes, stethoscopes, and ID badges. Three people are on their knees and four are standing behind them. The student added the caption, “Still laughing about all the times we cleaned BMs (Bowel Movements) off the floor,” followed by a smiling emoji.

Students shared artifacts that related to their exams or studying to their social media accounts. Participant 24 posted a picture that contains a small purple flower placed on top of a textbook page. The page is about the pathology and physiology of diabetes. The student added, “little guy brought me a flower while I’m studying” and included a red heart emoji. Several students posted memes that demonstrated how they perceive nursing exams. One meme read “Nursing school exams be like: The correct answer is POTASSIUM, not POTASSIUM. Some people put the second S before the first, which is not the most correct.” Another student shared a meme that read “How nursing exams be: The 75 yo male patient is suffering from COPD. His pulsox is 0%, his HR is 0, and his RR is 0. What would you, as the nurse, implement? (A) The patient is dead; (B) The patient is not living; (C) The patient is not alive; (D) The patient is deceased.”

Several participants shared content regarding their graduations, NCLEX experiences, and transition to practice. Four participants shared pictures of themselves wearing graduation gowns and posing by prominent institutional scenery. They captioned their posts with the sentiments of how happy they were to be finished their nursing education. Students also thanked their classmates for helping and supporting them throughout their programs. Participant 04 posted a picture of themselves in a graduation gown standing with two older women. The student posted, “Proud to announce I passed my NCLEX yesterday and will be joining these amazing women by becoming an RN.” Participant 03 tweeted about their success in obtaining their first nursing position in a celebratory tweet. The same student subsequently tweeted about some of their transition-related experiences and anxieties, saying “wow, being a new nurse is great! I love having anxiety after every shift cuz I’m afraid I did something wrong or forgot something (upside down smile emoji). Time to hit up my counsellor yo.”

Students shared content related to their extracurricular and informal learning activities. Two students shared several posts related to the Nursing Games, which appears to be a simulation and skills competition for nursing students. Participant 20 posted that “Nursing Games 2019 was a success! So proud of our team for placing 1^st^ in simulation and assessments, finishing 2^nd^ overall, and winning a compassionate care award!” The same student shared a summer learning opportunity that they were taking part in. The post was originally posted by the host health care organization. It read “Say hello to the future of nursing! These third year BScN students have been hired as [organization name] summer care providers and are celebrating nursing week with us today. Looking forward to seeing what this group gets up to over the next few months.” Other students shared artifacts demonstrating how they practised or performed their clinical skills. Participant 13 shared an Instagram story featuring an image of an orange with an open syringe. There is also a closed syringe in its packaging in the picture. The student captioned the picture “This orange will suffer so my patients don’t.” Another student posted about how they did cardiopulmonary resuscitation that day and another shared how they are proficient at starting intravenous lines. Finally, Participant 09 shared an award that they won during Nurses Week. The student posted a picture of flowers and a certificate that read ‘2019 Nurse of the Year’, recognizing the participant. The student described how “nursing is the hardest job I have ever done but I cannot imagine doing anything else. I am so grateful for this recognition and I wouldn’t be where I am today if it weren’t for my amazing mentors.”

Finally, numerous students shared content that related to the hidden curriculum of nursing education. Participant 19 shared a post originally posted to Facebook by a page called ‘I Love Nursing.’ The post read, “I don’t know who needs to hear this but a higher degree does not automatically make you a better nurse.” The student added:

I’m proud to be an RPN and my choice to obtain my degree is not to be a ‘better’ nurse. No book in the world can teach you how to be a compassionate, empathetic, caring person or how to devote your life to the care of others. Education does not equal quality. We’re all nurses and we all deserve respect and need to work together as a team.

Another student shared a meme that featured a looped video of Mr. Bean wearing a surgical mask and gloves and giving a thumbs up sign to the camera. The meme read “Problems in your personal life? Choose a medical career: No personal life, no problems!” Lastly, the same participant shared a tweet to Facebook that had originally been shared by a page called ‘IV League Tutoring for Nursing Students.’ It read “putting mental health before my education is a good idea until it affects my education which affects my mental health which affects my education.”

### Sharing educational tools, jobs, and resources

In this final category, participants posted content related to public health education topics as well as more specific nursing education tools and resources. A couple of participants also posted job opportunities to their social media pages.

Numerous participants shared posts relaying health information for their friends and followers on social media. Several students posted about changes in regulations for prescription medications in Ontario. One student shared an article related to changes in birth control availability and prescription processes; another student shared a Public Service Announcement (PSA) about testing for Sexually Transmitted Diseases (STDs). The STD PSA was written like an advertisement, trying to get the public to buy in to testing. Two participants posted health education posts about dementia and palliative care. The idea of both of these artifacts was to educate the public about how to handle family with dementia and what exactly palliative care is (myths vs. reality). For instance, one post featured what to do if living with someone with dementia. Strategies included not arguing with the person, diverting and distracting, reassuring the person, reminiscing with them, and encouraging them. Five posts provided educational content on what behaviours to expect from friends or children with mental illnesses, including anxiety and depression. Four posts were shared by the same participant (Participant 06) and focused on strategies for helping to calm a friend experiencing anxiety and what to have in a mental health crisis kit. Two participants shared the same post to Facebook about the value of seeking therapy.

Participants also shared content that advised their friends and followers what emergencies were appropriate for Emergency Department visits as compared to urgent care visits. For instance, one post provided a picture of a billboard in South Texas that advertised circumstances like “stepped on a bee—urgent care” on one side of the billboard and “stepped on a beehive—emergency care” on the other side. One participant provided a PSA with an explanation of what to expect from a hospital visit following a sexual assault. It outlined the timelines a patient might need to know in terms of starting medications to prevent STDs and pregnancy as well as deciding whether or not to report to the police. Lastly, two participants posted content relating to how to identify health-related pseudoscience.

Finally, the nursing student participants shared artifacts related to nursing tools, job opportunities, and other resources. One student shared three different nursing brain sheet templates to their Facebook page. One post describes nursing brain sheets as “report sheets, security blankets, flow charts, to-do lists, and everything in between.” The student encouraged their peers to post their brain sheet templates as well. One participant shared clinical pearls that they learned in the hospital, stating “nurse friends—learned something new today: called a Myxoma. Loose tumour within the heart causes a ‘plop’ sound on auscultation. Here’s the deets with video and link for audio” (Participant 08). Several participants shared details of continuing education opportunities like violence de-escalation workshops, disaster and emergency management, and wound management. Finally, participants shared job postings or career fair advertisements for nursing positions. Participant 19 shared an opportunity for a home care placement with a family that they had experience working with as a Registered Practical Nurse. Another participant posted numerous career fair opportunities for nurses alongside job postings for nursing position in long-term care and community health settings.

## Discussion

Social media can facilitate both formal and informal learning, support student engagement, and promote student interactions beyond the scheduled class time (Gagnon, 2015; Snodgrass, 2011). This engagement was evident in the participants’ use of social media as they shared content, commented on peers’ posts, and discussed aspects of their nursing education online. Moreover, the nursing students in this study frequently used social media for advocacy purposes, which is an essential component under CASN’s ‘leadership’ competency for baccalaureate nursing education. The literature supports the use of social media as a mechanism to learn advocacy and other intrinsic skills like collaboration and the nursing students in this study appeared to use social media to exercise their role as advocates amongst their friends and family members. Moreover, some students directly linked their advocacy-related posts to concepts they had learned in class or in clinical placements. This behaviour aligns with the literature on social media use in learning. For instance, Cole et al. (2017) found that students who participated in case-based learning (CBL) on social media demonstrated increased engagement when they began linking their learning and issues raised in the cases to wider health issues and contemporary affairs. Gagnon (2015) found that students agreed that using Twitter in their learning helped enhance collaborative relationships, increased their engagement with course content, and contributed to the quality of the course. Kind et al. (2014) argued that when used well, social media could increase engagement in learning by health professionals and enhance the public good. The advocacy and health education content shared by the participants to their own social media suggests that they are engaged with wider health and social issues and are able to draw connections between current events and classroom experiences.

The participants in the present study also shared content that explored their nursing identity and nursing roles. Some of this content was meme-based and reflected the expectations and realities of nursing practice while other content included professional development opportunities or news articles that explored key issues in nursing culture and work environments. In this way, social media appeared to play a role in shaping or informing nursing students’ emerging understanding of the nursing profession and their role(s) as a nurse. This finding is one that we did not find reflected in existing nursing education literature, which mainly focused on social media use in formal nursing education and by nursing faculty members. Some of the content shared by participants to their social media accounts featured memes depicting questionable clinician–patient interactions, such as the meme alluding to the perceived difficulties in educating so-called non-compliant patients. Marnocha and Pilliow (2015) indicated that risks to professionalism can include verbal or visual violations of patient confidentiality, unprofessional online content related to substance use and sexuality, and demeaning content about patients, peers, clinical sites, organizations, or instructors. Seemingly unprofessional content was the exception rather than the norm in this study, at least on participants’ personal social media pages; participants posted far more content related to advocacy or health education purposes as well as artifacts from their own formal and informal learning in nursing. While the extant literature on social media use in nursing education does focus on using social media to teach professionalism to nursing students, an interesting follow up study would be to explore how nursing students’ professional identities are shaped by the content they engage with in online nursing spaces, like through social media (i.e., the hidden curriculum of social media use).

This study found that nursing students share a range of content related to learning in nursing to their social media pages. While a few student posts could be perceived as unprofessional, the majority of content shared was related to advocacy, relaying health education, sharing artifacts from students’ own formal and informal nursing education, and exploring the identity and role of a nurse. Much of the existing literature on social media use in HPE focused on the platforms that students use for educational purposes or the content that faculty members share on social media for teaching purposes. Our initial literature review did not identify any articles that disseminated what educational content nursing students share with social media. A subsequent search of the literature following our study revealed analyses of content shared within medical education by practicing physicians or by educators for teaching purposes (Diller & Yarris, 2018; Nikolian et al., 2018; Riddell et al., 2019). Accordingly, the present study fills a gap in the literature by exploring the specific content that nursing students shared to their social media pages for learning purposes.

### Study strengths and limitations

This study consisted of a digital artifact collection wherein we followed nursing students’ personal Twitter, Facebook, and Instagram accounts over a period of 5 months to see what content they shared related to learning in nursing. Our methodological approach is fairly novel, especially in HPE research. We have not identified any studies that have conducted a similar digital artifact collection, where the study focused on following the same participants over an extended period of time to see what content they shared with their private social media accounts. Every published study that we encountered in the literature that explored what content students posted to social media was restricted to hashtag analytics over a shorter period, like during a conference or a course assignment (Junco et al., 2013; Sherbino, 2015; Sinclair et al., 2015). We also conducted a multimodal analysis and qualitative content analysis, which requires a high degree of researcher reflexivity. Since HPE—and health sciences more broadly—favor the hierarchy of evidence as a measure of research rigor and quality, other approaches like multimodal analysis as part of a larger study design are arguably less common (Evans, 2003; Mantzoukas, 2008).

A key limitation of this study was that we were unable to see what content the participants shared by Direct Message or in private social media groups. This limitation left the impression that seven participants did not share any content related to learning to social media during the study period. While the School of Nursing does not currently have their social media policy publicly available on their website, it is possible that students have been taught about or warned against using social media related to their nursing education and practice by their professors, thereby influencing what and how they post. Further, our presence as followers on the participants’ social media accounts may have influenced participants’ posting behaviour over the study period. Despite designing our study to mitigate any potential Hawthorne effect, the fact that participants may have changed their posting behaviour because they knew they were being observed remains a potential limitation to this study. Despite the aforementioned limitations, the findings of this study add valuable insights to the literature on what content nursing students share for the purposes of learning.

## Conclusion

Social media use in nursing education encourages students to make connections between content they encounter on their personal time and content they have seen in their nursing courses. It also has the potential to bridge several gaps in nursing education. Social media can allow students to discuss their clinical experiences with their peers— while being mindful of maintaining appropriate privacy and confidentiality—especially if they are feeling isolated or need support. Social media also allows nursing students to consolidate content from their classes, share resources ahead of their clinical rotations, and better anticipate the transition to practice as a new nurse. In many ways, the nursing students in this study already appear to be using them for these purposes. Importantly, social media provides a voice for nursing students to explore their professional identity and roles as nurse and advocate.
